# Supplementary material for: Fibrinolytic and antibiotic treatment of prosthetic vascular graft infections in a novel rat model
Source: PLoS One. 2023 Jul 18;18(7):e0287671. doi: 10.1371/journal.pone.0287671 (PMC10353806; doi:10.1371/journal.pone.0287671)
Supplement: S1 Fig — CLSM images of the biofilm formed at the vascular graft dissected crosswise (A) and lengthwise (B). Cells (bacterial and murine) are shown in blue (SYTO41) and fibrin is shown in green (anti-fibrin antibody, 59D8, Atto488 conjugated rabbit IgG). The 2D images are shown as two-channel. Scale bar 50 μm. (PDF) [file pone.0287671.s001.pdf]

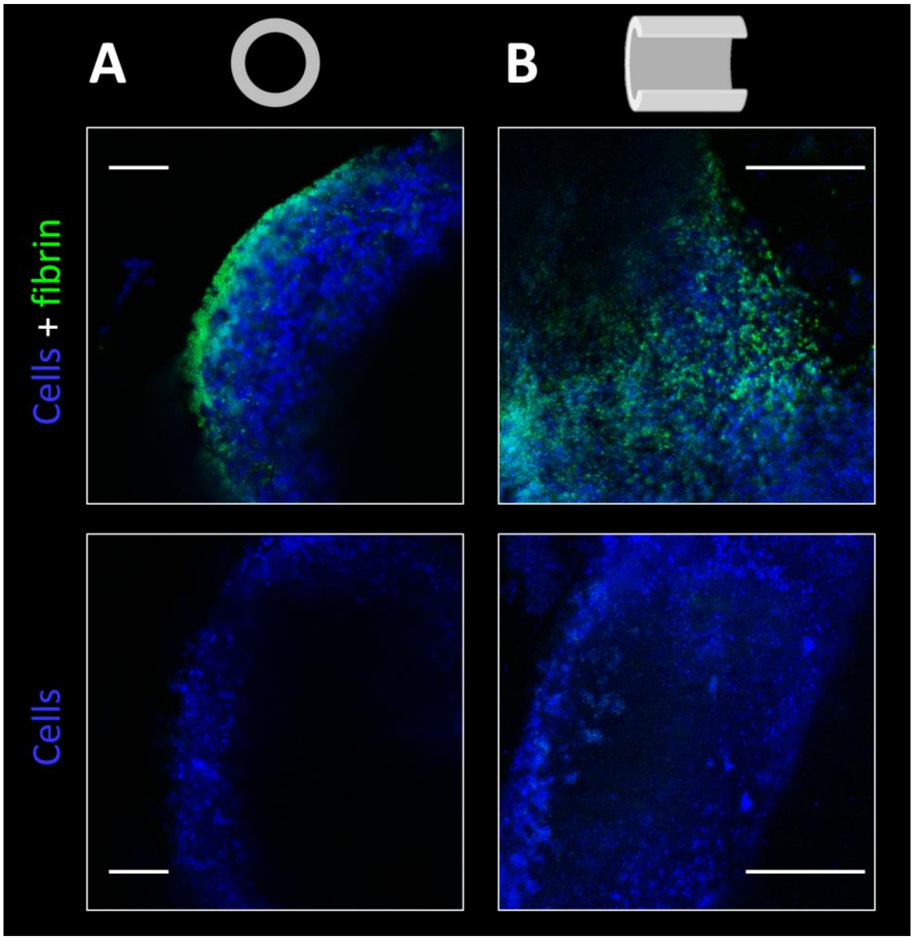

S2 Figure. Confocal laser scanning microscopy images of the MRSA biofilm formed in prosthetic vascular grafts in rats after 10 days of infection. CLSM images of the biofilm formed at the vascular graft dissected crosswise (A) and lengthwise (B). Cells (bacterial and murine) are shown in blue (SYTO41) and fibrin is shown in green (anti-fibrin antibody, 59D8, Atto488 conjugated rabbit IgG). The 2D images are shown as two-channel. Scale bar 50 µm.
